# Modified protocol for pterygium surgery without blades and electrocoagulation

**DOI:** 10.3389/fmed.2025.1522167

**Published:** 2025-02-21

**Authors:** Huimin Ge, Guofan Cao, Jian Wang, Shu Zhang

**Affiliations:** ^1^Affiliated Eye Hospital of Nanjing Medical University, Nanjing, China; ^2^School of Communication Science and Disorders, Dalhousie University, Halifax, NS, Canada

**Keywords:** pterygium, surgical removal, blunt separation, recurrence, electrocoagulation

## Abstract

**Purpose:**

To evaluate the efficacy, safety, and outcomes of a blade- and cautery-free surgical protocol for pterygium removal.

**Methods:**

Pterygium removal surgery was done in 69 eyes (67 patients; 24 males and 43 females) who were followed up for at least 6 months. The surgery was characterized by blunt separation of the pterygium from the head to the limbal arc using the tip of Vannas scissors and modified procedures, such as transpositional flapping and suture closure. Neither a blade nor a cautery was used.

**Results:**

The reported subjects were 60.7 years old on average, and most of them had primary pterygium (66 out of 69). The proposed surgical protocol was simple to perform, requiring an average operation time of 18.7 min which was shorter than that of the suture and fibrin glue groups mentioned in relevant reports. Post-anesthesia pain was relieved quickly 1 day after surgery without the use of pain killers. During the follow-up period of 11.3 ± 3.1 months, recurrence of pterygium requiring additional surgery was seen in only three eyes (4.3%).

**Conclusion:**

The potential of the examined protocol as an easy, efficient, and reliable approach was demonstrated.

## Introduction

Pterygium is a wing-shaped overgrowth of fibrocytes on the ocular surface with fibrovascular conjunctival proliferation and/or inflammation, which can potentially grow up to the corneal surface (1). It is presumably due to ultraviolet-induced damage to the limbal stem cells (2). Surgical removal is indicated for both visual and cosmetic reasons. However, there is no guideline or consensus on the surgical protocol and the management of this disease.

Although surgical removal of pterygium is relatively easy, significant efforts have been made to optimize it. Traditional surgery starts by separating the pterygium from the limbal arc, and this step is usually done with sharp scissors or surgical blades (3–5). Sophisticated technologies, such as phototherapeutic keratectomy (6, 7) and low-temperature plasma (8) have been adopted into the surgery. However, the outcomes are often unsatisfactory, as evidenced by residue pterygium tissue on the cornea and/or uneven corneal surface that requires further scraping, a procedure that can cause corneal damage (7).

In the present report, we used a protocol containing two major modifications: 1. blunt dissection starting from the pterygium head, and 2. not using cautery. Two video demonstrations are provided in the Supplementary material of this report to demonstrate the feasibility of our new protocol.

## Methods

This retrospective study included the patients who underwent pterygium surgery by our team between January and September 2023. Consent was obtained from each individual subject for the use of data in this report, and the study was approved by the Affiliated Eye Hospital of Nanjing Medical University and conducted according to the Declaration of Helsinki.

The cases were recruited with pterygium size ranged from less than one-third of the corneal diameter to those spread into the pupillary area, which were also graded according to its position of the advancing edge related to the corneal diameter. Cases with concurrent ocular surface pathology including severe dry eye, symblepharon and blepharitis were excluded. All recruited cases were followed up for at least 6 months. Data collected included demographic information, medical history, preoperative evaluation, surgical time, pain level during and 1 day after surgery postoperative course, aesthetic appearance of the surgical area, and complications that might occur.

### Surgical technique

All surgeries were performed by one surgeon at the Affiliated Eye Hospital of Nanjing Medical University. After topical anesthetics, the conjunctiva over the pterygium was then ballooned using lidocaine mixed with epinephrine (lidocaine hydrochloride 2% and epinephrine 1:100,000). The neck of the pterygium was pulled up by using a forceps and then the pterygium was bluntly dissected by using a Vannas scissors. The scissors was closed so that the tip (not the sharp edge) was inserted from the arc edge between the pterygium and the cornea surface and extended both superiorly and inferiorly towards the margin near the plica semilunaris. The scissors tip was then inserted deeper to separate head of the pterygium towards the limbus until the pterygium is completely free (Supplementary Video S1). This procedure exposed the entire fibrovascular stalk of the pterygium. The remaining fibrovascular tissue over the medial rectus insertion was further torn off and removed.

A superior conjunctiva flap (approximately 5.0 × 5.0 mm) was obtained from the fornix to the limbus. This flap was used to cover the surface of limbal area exposed after the pterygium removal. Ballooning of conjunctiva and Tenon’s capsule facilitated the splitting of a thick layer of the flap. The residual layer of Tenon’s capsule was maintained at the donor site to prompt conjunctivalization. The flap was obtained by cutting the limbal area and was used to cover the bare sclera. The limbus edge of the flap was placed to face the limbus. The flap was fixed in the wound using 5-6 interrupted sutures with 10-0 nylon at each corner, and an additional suture was placed at the limbus of the cornea (Supplementary Video S2). Sharp instruments (such as blade) and bipolar cautery were never used during all surgical procedures.

All nylon sutures were removed 1 week after the surgery. The postoperative medication included a topical antibiotic and steroid drops four-time daily for the first week and then tapered down every week by one drop for 1 month, followed by non-steroidal drops twice a day for 4 weeks. Regular postoperative ophthalmic examinations were conducted for 1 week and then each month till 6 months.

### Outcome measures

#### Clinical classification of pterygium (8)

Pterygium was graded according to its position of the advancing edge related to the corneal diameter.

**Figure 1:**
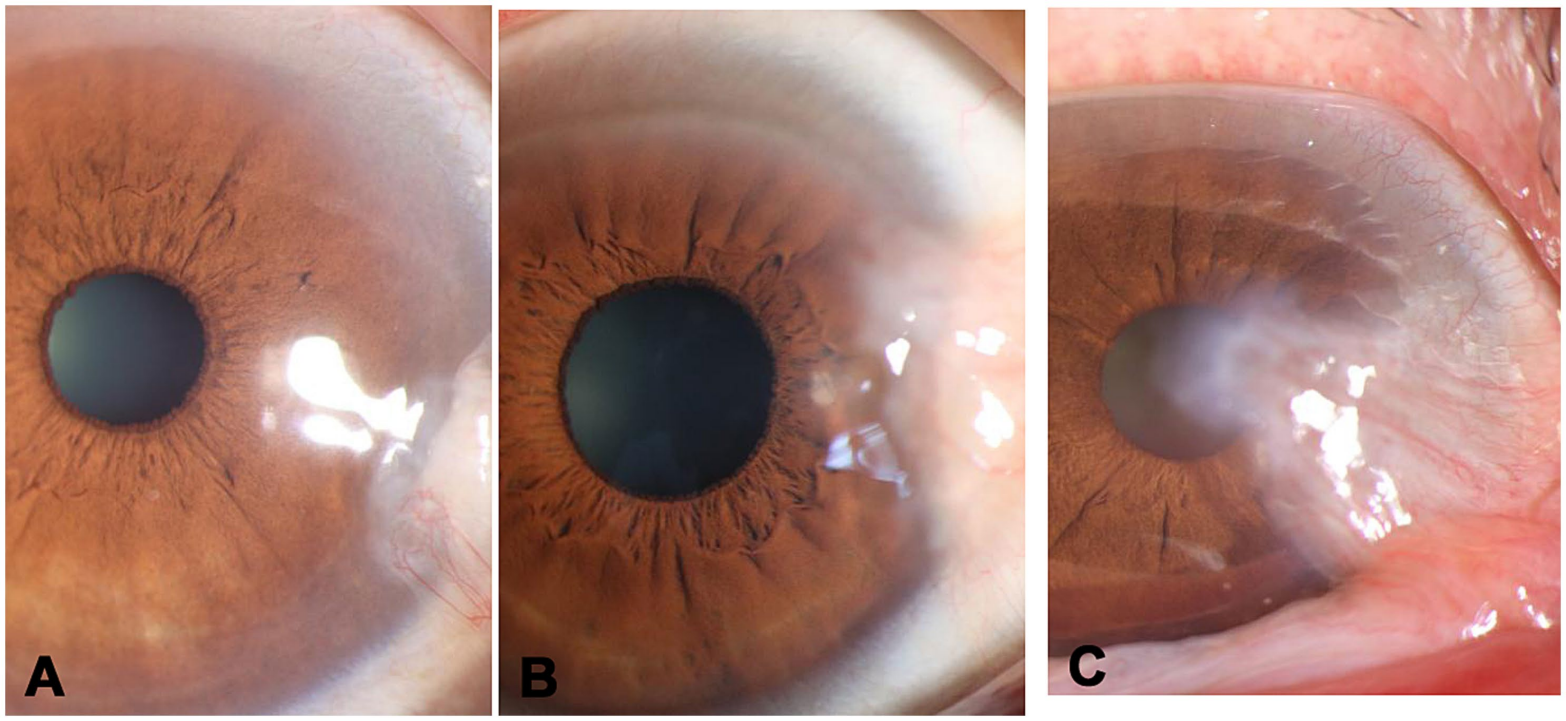
Clinical classification of pterygium. **(A)** grade 1-less than 1/3 of the corneal diameter, **(B)** grade 2-between one-third of the corneal diameter and the edge of pupil, **(C)** grade 3-within the pupillary area.

G1, less than one-third of the corneal diameter (Figure 1A).
G2, between one-third of the corneal diameter and the edge of pupil (Figure 1B).
G3, within the pupillary area (Figure 1C).

#### Pain score

Degree of pain was evaluated by a subjective ranking method described previously (9, 10), which ranks the pain in four levels:

0, no pain (no sensation).
1, little pain (only sensation).
2, moderate pain.
3, severe pain.

The pain score was ranked by each patient immediately after surgery (for the pain during surgery) and repeated 1-day post-surgery. Standard instruction was used in the ranking to avoid bias.

#### Surgical time

All surgeries were recorded on video for observation of surgical time, which was measured starting from the placement of the lid retractors to the removal at the end of surgery (8).

*Postoperative grading of the cornea* on the basis of slit lamp examination and front segment photo (3).

Score examination findings 1 week of the postoperative cornea.

**Figure 2:**
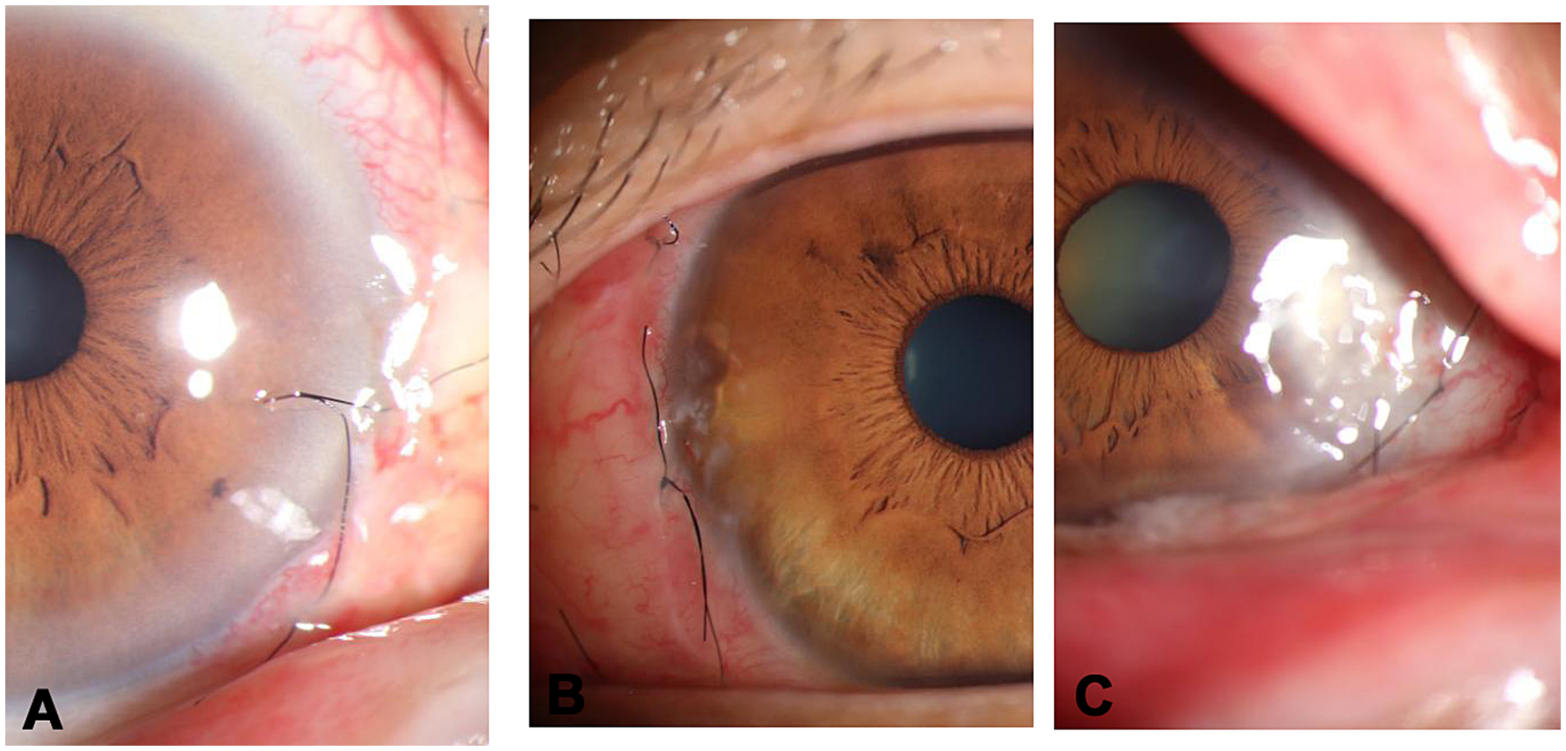
Postoperative grading of the cornea. **(A)** grade 1-clear, **(B)** grade 2-mild keratitis, **(C)** grade 3-clouding at surgical site.

Grade 1, clear (Figure 2A).
Grade 2, mild keratitis (Figure 2B).
Grade 3, clouding at surgical site (Figure 2C).
Grade 4, lamellar fill.

*Graft stability* was observed based on the number of sides displaying gaping/displacement in the graft-bed junction (10).

*Recurrence* was defined by fibrovascular regrowth extended to the cornea across the limbus (11).

## Results

Totally 67 patients (24 males and 43 females; age of 60.7 ± 11 years) were recruited into this report. The surgery was done on 69 eyes, 66 (96%) of them with primary pterygium and 3 (4.3%) eyes with recurrent pterygium. Clinical classification of pterygium: G1, 11 eyes; G2, 36 eyes; G3, 22 eyes. During the follow-up period of 6 months, recurrence was seen in three eyes (4.3%).

Slit lamp and visual examination were performed by the surgeon and photographically recorded at the operative site for all patients on postoperative day 7.

The pain score during and 1 day after surgery are shown in Table 1.

**Table 1:** Pain scores as indicated by the number of eyes.

Pain scores	0	1	2	3
During operation (eyes)	2	62	4	1
One day post-operation (eyes)	1	3	54	11

*p < 0.001.*

Table 2 shows the postoperative grading of the cornea on the basis of slit lamp examination and front segment photo.

**Table 2:** Postoperative grading of the cornea on the basis of slit lamp examination and front segment photo.

	Grade 1	Grade 2	Grade 3	Grade 4
Eyes	13	49	7	0

Postoperative grading 1 and 2 of the cornea was achieved in 90% cases.

**Figure 3:**
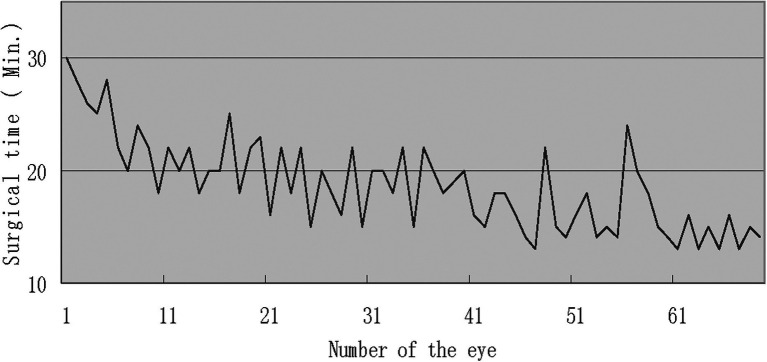
Surgical duration of each patient in order.

Figure 3 records the surgical duration of each patient in order.

Follow-up period: from 11.3 ± 3.1 months. No graft dislocation occurred. No postoperative complications such as conjunctival granuloma, diplopia, and ocular hypertension were found in any case. No scleral thinning or melting was encountered.

## Discussion

Clear removal of the pterygium from the cornea is a key step for the success of the surgery, since the unexcised abnormal fibrovascular tissue is the reason of pterygium recurrence (8, 12). In addition, inappropriate dissection may cause cornea damage and roughness, which is also a risk factor for recurrence. Traditionally, surgical blades and other sharp instruments are used for separating the head of the pterygium from the cornea (13). Recurrence and rough corneal surface were often reported after such surgery. Many sophisticated technologies have been proposed and used to overcome this problem, including the use of diamond burr to smooth the corneal surface (7), the use of phototherapeutic keratectomy to ablate all visible residual tissues (6) and the use of low-temperature plasma for excision and hemostasis (8). However, those methods were either expensive or difficult to perform; and the outcome was inconsistent, based on our experience.

Since the pterygium head and the cornea are two different tissues, the connection between them is likely to be weaker than that within each tissue. This was our initial reasoning for the use of blunt dissection. Moreover, separating the pterygium from the edge of the head of the pterygium towards the neck is easier since it is under direct vision to allow a correct separation. As soon as this initial separation was established, the extension was easy (Supplementary Video S1).

Several advantages of our surgical protocol were seen in this report and examined below.

Firstly, the ablation by blunt surgery is more thoroughly completed, leaving the surface without visible subepithelial fibrovascular tissue. Blunt dissection allows a single dissection plane all the way to the fornixes. The single dissection plan and avoiding abrupt cutting help maintain bloodless field throughout the procedure. This clean removal is likely the reason for the lower recurrence rate of this report. The recurrence rate was 4.3% in the present report (three cases in 69 eyes), which was in the lower end among the previously reported rate of 0–20% (1, 8, 14). A comprehensive comparation is impossible since the recurrence rate is influenced by many factors that are beyond control (15). Among the three cases of recurrence, two cases were found to have dry eye syndrome as indicated by abnormal results of examinations, which were provided with further clinical observation and medication treatment.

Secondly, the blunt separation leaves a smoother corneal surface. The wound left after the pterygium removal was minimal. This largely reduced the need for surface smoothing after the removal, speeded up the repair of corneal epithelium and reduced the scar formation. This advantage was linked to better cosmetic outcome. In addition, the blunt dissection leads to minimal bleeding, so that the use of adjunctive agents, such as blade cutting, mitomycin C and electrocoagulation (16, 17) was abandoned. This not only reduced surgical trauma and postoperative reactions, but also shortened the surgical time. Traditionally, electrocautery pens or disposable cautery has been the most frequently used devices for hemostasis in pterygium surgery. However, cautery generates a direct current to heat a wire loop at the end of the device up to 350°C to 400°C, leading to acute tissue damage and wound ischemia and even delayed wound healing and inflammatory reactions (8, 18).

Bare sclera technique is the first technique adopted for pterygium removal and is characterized by simple excision, but unfortunately, which resulting in high recurrence rates. Then we realized that the damage to the limbal stem cells cause focal conjunctivalization of the cornea (3). So covering the bare sclera has been shown to significantly reduce not only postoperative pain and inflammation (17), but also recurrence rate. The methods include anchored conjunctival rotation flap, autologous conjunctival tissue and amniotic membrane.

The application of amniotic membrane and anchored conjunctival rotation flap appear to be safe and effective, and it is associated with lower recurrence rates when compared with the bare sclera technique. However, when compared with conjunctival transpositional flap, amniotic membrane efficacy remains controversial (19).

Therefore, we adopted free conjunctival flap transplantation, if there is sufficiently healthy conjunctiva, which not only ensures better alignment of the wound edge, but also keeps the growth and distribution direction of corneal limbal cell tissue (including stem cells) consistent with the original. For pterygium surgery, pterygium excision with conjunctival transpositional flap is the gold standard procedure, which has been linked to the least risk of recurrence (20, 21).

Suture is needed to fix conjunctival grafts on the scleral wound, which requires skill and is time consuming. Recently, some alternative suturing methods have emerged in the attempt to improve the outcome (10). However, we think that the suture method with 10-0 nylon suture adopted (Supplementary Video S2) in the present study is more reliable. No dislodgement of graft was encountered.

All of the surgeries were performed by a single surgeon (HG), a 3rd year resident surgeon trained at our hospital, completing five cases of assistants in the surgery reviewed in the present report (within 1 month). After completing half of the surgery in this sample, the surgical duration was reduced to around 15 min, which was shorter than the duration reported by others in which suture or even the fibrin glue was used in fixing the grafts (8, 12, 14). The mastery level of the surgery is demonstrated by Figure 3 which shows the time length of the surgery and the learning curve.

Appropriate anesthesia during surgery made patients feel less pain, and the use of anesthesia ballooning the conjunctiva to separate the conjunctiva and Tenon’s layer also alleviated the patient’s pain. Therefore, the pain score during surgery is lower (Table 1). As the anesthetic effect disappears, patients gradually felt the pain. The postoperative pain was mostly caused by a foreign body sensation due the corneal wound. As the corneal epithelium healed within 24–48 h after surgery, the pain was significantly relieved. Advising patients to reduce eye movement after surgery can also alleviate pain.

In conclusion, this study clearly shows that the blunt dissection is more suitable for the separation of pterygium from the cornea since they are two different tissues. Compared to using blade, blunt dissection show less bleeding and reduced the chance of damaging to surrounding tissue, making cautery unnecessary. This is also associated with the shorter surgical duration.

### Limitations

A larger number of clinical trials may be necessary to validate our blunt dissection of pterygium technique and personalized it with other parameters in the prospective controlled studies (22).

## Data Availability

The original contributions presented in the study are included in the article/Supplementary material, further inquiries can be directed to the corresponding author.

## Supplementary material

Supplementary VIDEO S1 Blunt separation of pterygium head and cornea.

Supplementary VIDEO S2 Adopting suturing to fix the transplanted flap.
